# Peripheral Ameloblastoma: A Case Report and Review of Literature

**DOI:** 10.1155/2012/571509

**Published:** 2012-04-09

**Authors:** V. T. Beena, Kanaram Choudhary, R. Heera, R. Rajeev, R. Sivakumar, K. Vidhyadharan

**Affiliations:** Department of Oral & Maxillofacial Pathology, Government Dental College, Trivandrum, Kerala 695011, India

## Abstract

Peripheral ameloblastoma, a rare and unusual variant of odontogenic tumour, comprises about 2–10% of all ameloblastomas. The extraosseous location is the peculiar feature of this type of tumour, which is otherwise similar to the classical ameloblastoma. This paper describes a case of peripheral ameloblastoma in a 67-year-old female affecting the lingual alveolar mucosa of the mandibular 32–34 region which was clinically diagnosed as pyogenic granuloma. This paper becomes important due to availability of all data, makeing it a well-documented case.

## 1. Introduction

Prevalence of odontogenic tumor is 0.8% of all oral and maxillofacial pathology. Ameloblastoma contributes 30% of all odontogenic tumor [1]. The peripheral ameloblastoma (PA) is an exophytic growth localized to the soft tissues overlying the tooth-bearing areas of the jaws, the initial diagnosis often being fibrous epulis. In most cases there is no radiological evidence of bone involvement, but a superficial bone erosion known as cupping or saucerization may be detected at operation. The overall average age is 52.1 years, slightly higher for males (52.9 years) than for females (50.6 years). Thus, the PA occurs at a significantly higher age than the intraosseous ameloblastoma (IA-37.4 years). The male/female ratio is 1.9 : 1, as opposed to 1.2 : 1 for the IA. As to the location of PA, the maxilla/mandible ratio is 1 : 2.6. The mandibular premolar region accounts for 32.6% of all sites [2]. Here, we are reporting a case of peripheral ameloblastoma arising from mandibular 32–34 region, clinically diagnosed as pyogenic granuloma. 

## 2. Case Report

**Figure 1:**
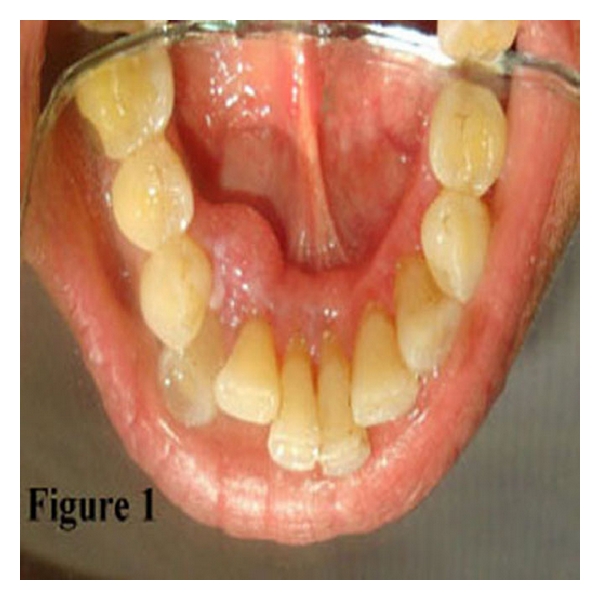
Swelling at lingual alveolar mucosa of the mandibular 32–34 regions.

**Figure 2:**
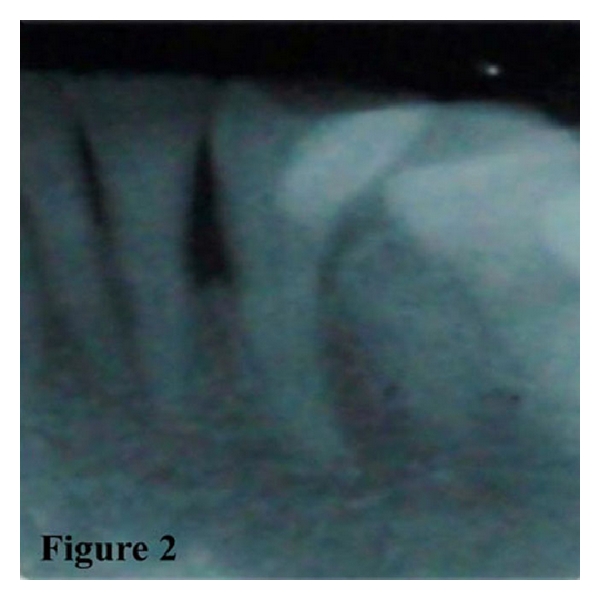
Radiograph showing horizontal bone loss between 32–34 regions.

**Figure 3:**
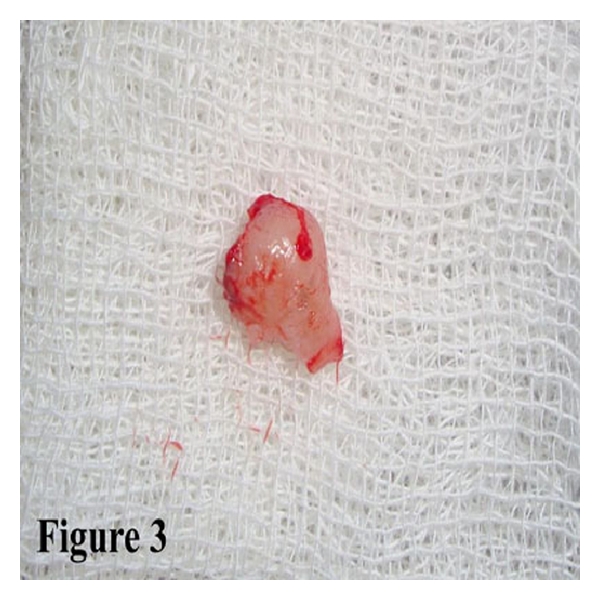
Gross specimen.

A 67/F patient reported to the Periodontia Department of Government Dental College, Trivandrum, Kerala, India, with a chief complaint of swelling on the left anterior lingual aspect of the mandibular gingiva of 1-year duration. There was no history of trauma or infection. Intraoral examination showed a well-demarcated round, firm, nontender, and sessile growth with smooth surface, extending from 32 to 34 regions lingually and measuring (1.5 × 1.5) cm in diameter. The colour of the swelling was the same as that of adjacent mucosa (Figure 1). There was no bleeding on probing, and involved teeth were vital. Radiographically, there was horizontal bone loss between 32-33 (Figure 2). Oral hygiene status was good. Clinical diagnosis was made as pyogenic granuloma. The growth has been excised under local anaesthesia with proper antiseptic measures. The excised tissue sent for histopathological examination. Gross specimen was a single bit of size (1.2 × 0.6 × 0.4) cm, brown in color, and firm in consistency (Figure 3).

**Figure 4:**
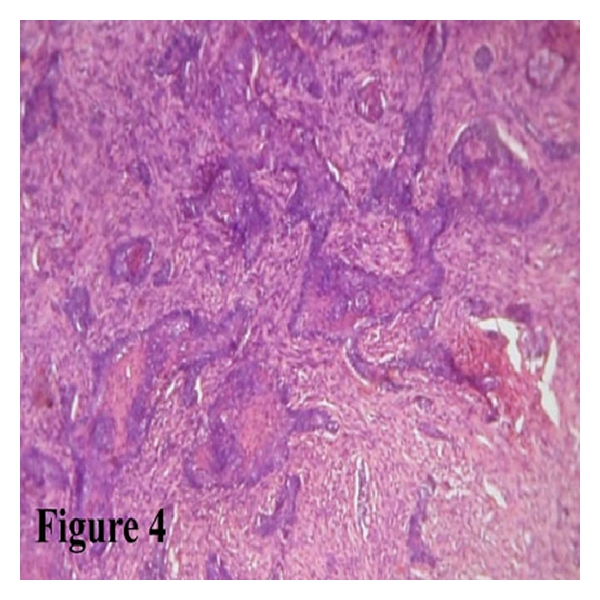
H and E-stained section shows ameloblastic follicles with basaloid pattern (10×).

**Figure 5:**
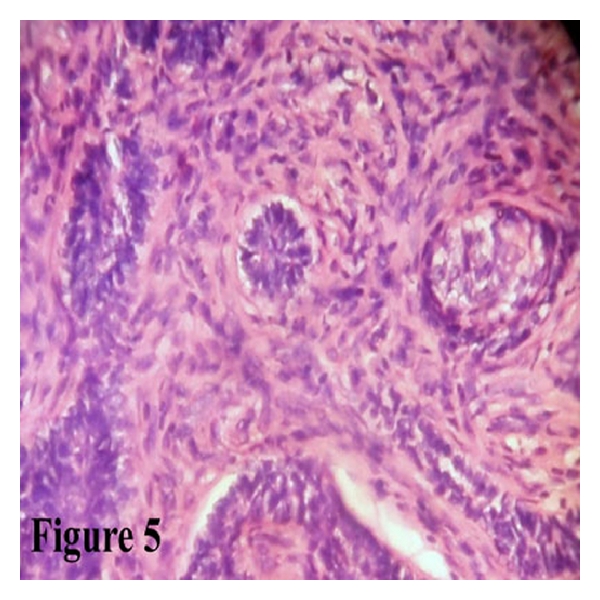
H and E-stained section shows ameloblastic follicles with acanthomatous changes (40×).

Microscopy showed surface epithelium, and deep in the lamina propria numerous odontogenic follicles were present in a cellular and moderately collagenous connective tissue background. Most of the follicles were arranged in a basaloid pattern (Figure 4) and showed acanthomatous change (Figure 5). A diagnosis of peripheral ameloblastoma was made.

## 3. Discussion

 The PA is a rare, benign, and extraosseous odontogenic soft-tissue tumor that was first reported in the literature by Kuru in 1911. But Stanley and Krogh's case reported in 1959 is considered by some authors to be the first well-established and true case of peripheral ameloblastoma [3]. 

 The pathogenesis of PA has been discussed extensively with most probable source of this lesion is remnants of the dental lamina, the so-called “glands of Serres,” odontogenic remnants of the vestibular lamina, pluripotent cells in the basal cell layer of the mucosal epithelium and pluripotent cells from minor salivary glands [4]. 

 Immunohistochemically, PA showed positive reactivity for AE1/AE3, KL1,34, E12, and MNF116 cytokeratin and negative staining for CK8, CK10, CK13, CK17, and CK18, are in accordance with the observation seen in human enamel organ [5]. Ameloblastomas, whether central or peripheral, are essentially devoid of CK7, CK8, CK10, CK18, CK20 [4, 6, 7], and epithelial membrane antigen [8]. 

 Almost similar ultrastructural features were described by different authors include three different zones, namely, the deep tumor islands, the “altered” surface epithelium overlying the tumor, and the transitional area between “normal” and “altered” covering epithelium. The inability to identify, on an ultrastructural basis, the presence of a transitional “preameloblastomatous” zone, either in the altered epithelium or laterally at the junction between altered and normal gingival epithelium, precludes any definitive elusions on matter whether the tumor is derived from the oral mucosa or from remnants of “odontogenic” epithelium within the underlying connective tissue. Ultrastructural features were also similar to central ameloblastoma [5].

 It was concluded that although both tumours have similar histomorphological characteristics, their clinical appearance and behaviour were completely different. The PA is slow growing and noninvasive, and recurrence is uncommon following excision, in contrast central ameloblastoma, is locally invasive and can destroy large segments of the jaw. Dense fibrous tissue of the gingiva and periosteum and the cortical plate of the alveolar process may be responsible for an effective barrier to the infiltration of peripheral ameloblastoma [9].

 It has also been reported at unusual site like buccal mucosa [4], base of tongue [10], and floor of the mouth [11], regardless common site being mandibular lingual gingival.

 PA may exhibit various histological patterns as found in the intraosseous ameloblastoma. It has a marked tendency to be acanthomatous as seen in our case. However granular cell [12] and desmoplastic [6] variant of peripheral ameloblastoma have also been reported.

 PA should be differentiated from peripheral reactive lesions such as pyogenic granuloma, epulis, papilloma, fibroma, peripheral giant-cell granuloma, peripheral odontogenic fibroma, peripheral-ossifying fibroma, Baden's odontogenic gingival epithelial hamartoma, and basal cell carcinoma [2]. Positive immunohistochemical staining for Ber-EP4 in lesion provides strong evidence for the diagnosis of basal cell carcinoma [7].

 PA is usually a benign, slow-growing tumor with no invasive potential. Later, Buchner and Sciubba reported 9% of recurrence following treatment [13]. Though malignant transformation is rare, metastasis has also been reported [14].

 The current treatment of choice is conservative supra periosteal surgical excision with adequate disease-free margins [2]. Continuous followup is necessary as late recurrence is also reported. In our case, complete surgical excision was done, and patient is on continuous followup since last 4 months with no recurrence noted so far.

 In conclusion, PA is an uncommon odontogenic neoplasm. As the isolated basal cell carcinoma of the oral cavity has also been reported [7], a careful histopathological evaluation is necessary to differentiate PA from basal cell carcinoma of oral cavity, and all possible cases should be evaluated by immunohistochemistry. Throughout, microscopic examination of the specimens are needed to ensure that the margins are clear of the tumor. Treated cases of PA should be followed up for a longer duration to detect the late local recurrence.
